# Salvage surgical procedure for artificial sphincter extrusion

**DOI:** 10.1590/S1677-5538.IBJU.2017.0462

**Published:** 2018

**Authors:** Flavio Trigo Rocha, Jean Felipe Prodocimo Lesting

**Affiliations:** 1 Centro de Incontinência Urinaria, Hospital Sírio Libanês, SP, Brasil

**Keywords:** Urinary Incontinence, Surgical Procedures, Operative, Urinary Sphincter, Artificial

## Abstract

**Case Hypothesis:**

Surgical removal is the standard treatment for artificial sphincter extrusion. However in some specific situations is possible to maintain the prosthesis with good results.

**Case report:**

We report a 60 years old patient presenting sphincter pump extrusion one month after artificial urinary sphincter (AUS) AMS 800™ placement for treating post-radical prostatectomy urinary incontinence (PRPUI). He also had a penile pros- thesis implant one year before that was replaced in the same surgery the sphincter was implanted. As patient refused sphincter removal and there were no signals of active infection he was treated by extensive surgical washing with antibiotics and antiseptics. Pump was repositioned in the opposite side of the scrotum. Patient had good evolution with sphincter activation 50 days later. After 10 months of follow up, patient is socially continent and having regular sexual intercourse. Savage surgery may be an option in select cases of artificial sphincter extrusion.

**Promising future implications:**

Like in some patients with penile prosthesis some pa- tients with artificial sphincter extrusion can be treated without removing the device. This may be a line of research about conservative treatment of artificial sphincter complications.

## SCENARIO

We described the case of a 60 years old male submitted to Radical Prostatectomy 8 years ago. All the surgical margins were negative and postoperative PSA remain lower than <0.003ng/dL. The patient also had diabetes type II and hypertension under good clinical control. Since the catheter removal patient presented severe urinary incontinence and severe erectile dysfunction. Pelvic floor rehabilitation was attempted without success. Patient had a continuous leakage during the day. During the night he improved continence and was able to void twice a night with a good urinary stream even though he needed to use one pad/night. Urodynamics showed a good bladder capacity with a Valsava leak point pressure of 35 centimeters of water. He was not able to elaborate a voiding diary once he leaked most part of the day.

After one year, he was treated by artificial sphincter placement with an improvement of 80% in continence (he reduced the number of pads/day from 6 to one or two). However, he remained wearing 1-2 pads a day and he was unsatisfied with his quality of life.

After 2 years, a second cuff placement was attempted and patient developed urethral fistulae requiring removal of all the artificial sphincter system. The fistulae were treated by a silicone Foley catheter placed in the urethra during 14 days.

Six months later, he underwent a new sphincter placement associated to semi rigid prosthesis implantation in order to treat the incontinence and the erectile dysfunction. He had new urethral extrusion requiring all artificial sphincter system removal but remained with the penile prosthesis.

Patient remained totally incontinent wearing up to 10 pads a day. Patient also referred no sexual activity due to incontinence.

Urodynamic evaluation showed severe sphincter deficiency (Valsalva leak point pressure = 45cmH_2_O). Cystourethrogram showed no urethral stenosis.

He was then submitted to new sphincter placement using a transcorporeal cuff. Urethral integrity was confirmed by an urethrocistoscopy carried out just at the beginning of the procedure. The surgery was uneventful but during the surgery we had to implant the pump through a scrotum incision due to extensive local fibrosis caused by previous surgeries.

**Figure 1:**
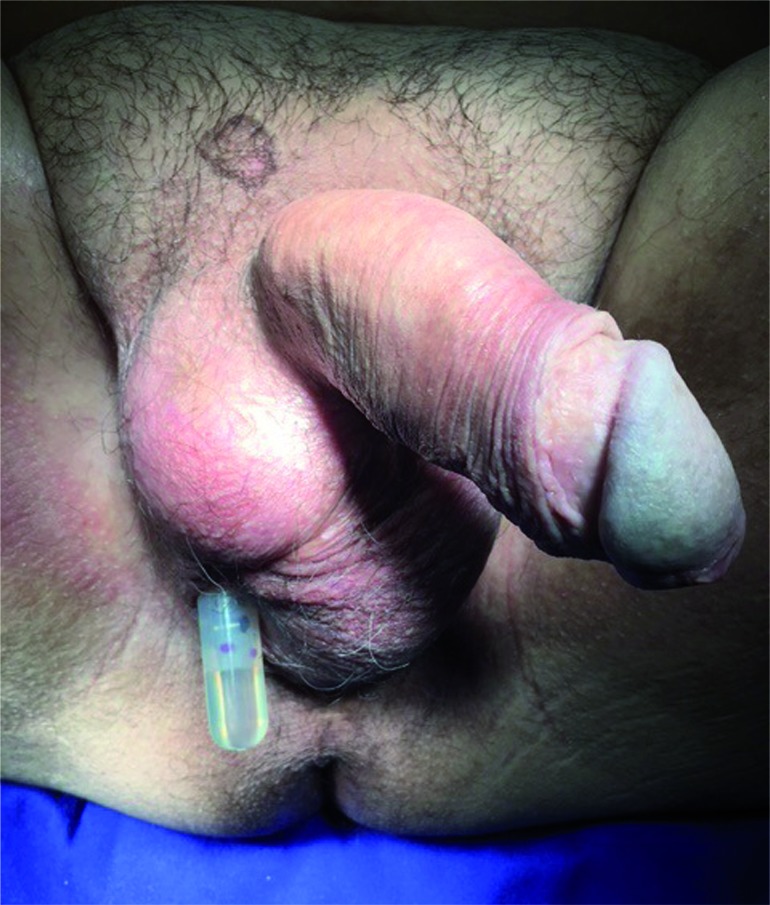
Initial aspect showing the pump extrusion through the scrotum skin. Note the absence of inflammatory signs or secretion.

One month after this new intervention, patient developed pump extrusion through the scrotum. There was just a discrete local secretion and no signs of systemic infection (Figure-1).

As the patient refused the standard treatment (removal of all system) we took him to operating room and under general anesthesia we carried out an extensive wound clean with antibiotics solution (cephalosporin and gentamicin solutions) as well as with chlorexidine solution. In addition, we did a surgical removal of all inflammatory tissue around the pump tubes and moved the pump to the opposite side of scrotum (right side). Patient received large spectrum IV antibiotics (Ceftriaxone plus vancomicin) during 3 days. After that, he was discharged with oral ciprofloxacin during 14 days.

Patient had a good evolution, without signals of local or systemic infection attested by local examination, urinalyses, hemogram and PCR. The sphincter was activated after 8 weeks.

**Figure 2:**
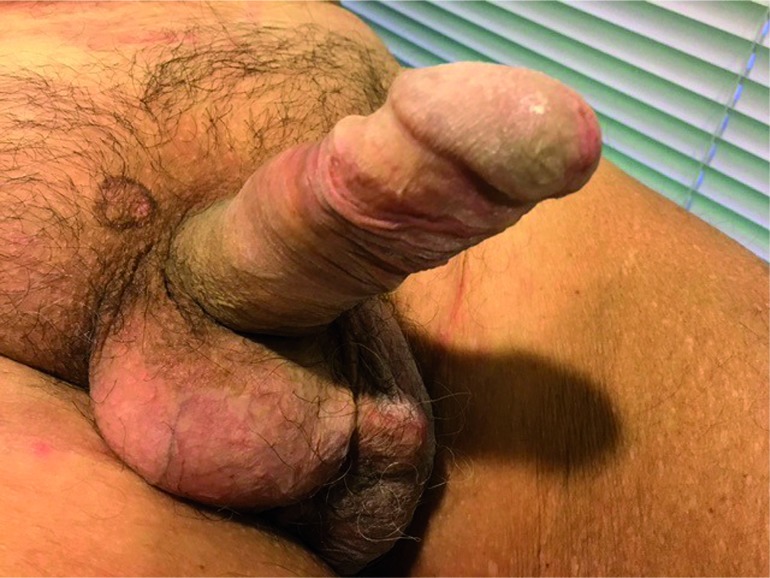
Ten months of follow up after the last surgery. Note the pump under the skin in the left side and no signals of infection or erosion.

Currently, after 14 months of follow up, the local aspect is excellent (Figure-2) and blood and urine tests are normal. Patient is socially continent, wearing one pad a day and resumed his sexual life resulting in a great quality of life.

## CASE(S) HYPOTHESIS AND RATIONAL

Urinary Incontinence is the most devastating long term complication of radical prostatectomy in terms of patients' quality of life (1).

The artificial urinary sphincter AUS is considered the gold standard treatment for this condition (2). The most representative series evaluating the artificial sphincter AMS 800 for the treatment of PRPUI shows this procedure is effective in almost 90% of the patients suffering from post prostatectomy urinary incontinence (3). The efficacy of this method can be summarized in Table-1 (4). However, complications may occur in about 15% of the patients treated with this proce-dure. These complications include mechanical failure in 5%, erosion in 5% and infection in another 5% (4). Table-2 summarizes these data in different publications (4). In cases of mechanical failure the broken part or the whole system can be removed and replaced in the same procedure. On the other hand, in cases of erosion and infection, the standard treatment is the sphincter removal and new sphincter implantation after three or more months. The rational for this approach is that removal of the synthetic components allows the antibiotics together with immunological system to eliminate all the bacteria and allowing a safe new implantation. However, patients submitted to sphincter removal due to erosion have a higher complications rate and a higher chance of new erosion (5).

**Table 1:** Efficacy of the artificial sphincter AMS 800 in different series in literature.

Author	Year	Number	Follow-up (years)	Continence rate (%)
Marks; Light, (6)	1989	37	3.0	94.5
Montague et al., (7)	1992	166	3.2	75.0
Perez; Webster, (8)	1992	49	3.7	85.0
Light; Reynolds, (9)	1992	126	2.3	96.7
Martins; Boyd, (10)	1995	28	2.0	85.0
Fleshner; Herschorn, (11)	1996	30	3.0	87.0
Mottet et al., (12)	1998	96	1.0	86.0
Trigo-Rocha et al. (4)	2008	40	2.5	90.0

**Table 2:** Complications of the artificial sphincter AMS 800 in different series of the literature.

Series	Year	Number	Infection (%)	Erosion (%)	Mechanical failure(%)
Gundian et al. (13)	1989	117	2.5	7.0	16
Marks; Light (14)	1989	16	5.4	8.1	NR
Litwiller et al. (15)	1996	65	6.0	3.1	NR
Singh; Thomas (16)	1996	28	10.0	0.0	NR
Elliot; Barrett (17)	1998	160	1.8	1.0	9
Trigo-Rocha (4)	2008	40	2.5	5.0	5

Penile implant represents another example of prosthetic material implanted to restore a phy-siologic function. They were introduced decades ago as a treatment of erectile dysfunction. Infection and/or erosion associated with placement of any prosthetic material are feared complications and the standard treatment is its prompt removal. An option, termed a salvage (or rescue) procedure, is cleansing the wound with a series of antiseptic solutions and replacing a new prosthesis during the same procedure. The other alternative is to return at a later date to replace the implant. However, the latter approach is associated with technical difficulties for insertion the implant mainly due to local fibrosis. Most patients elected the salvage approach because they were highly motivated to continue sexual activity obtained with the implant placed initially. Mulcahy first described the salvage procedure for penile implant infection. His objective was to avoid difficult revision surgery, penile shortening and patient discomfort (6). Success rate of this procedure could be high as 84% (7). In patients with postoperative purulent penile prosthesis infection and no evidence of systemic infection, a conservative “local rescue” without explanation was also described. These authors employed a conservative treatment strategy by local and systemic application of clindamycin before resorting to surgical exploration with or without salvage procedures (8). Following the same principles, a group described 8 patients with an infected artificial urinary sphincter who underwent a total of 9 salvage operations. In a 33 month mean follow-up, 7 patients were free of infection with a functioning artificial urinary sphincter. In one patient, the system was removed 16 months later secondary to urethral erosion. They concluded that salvage and immediate new implantation of an infected, non eroded single or double cuff artificial urinary sphincter appears to be a valid option with 87% overall success rate. These authors also pointed out that an associated inflatable penile prosthesis does not prohibit si-multaneous salvage of the two devices (9).

However, these authors did not try salvage procedure in any patient with sphincter erosion. There are in the literature two cases describing cuff erosion left without treatment (10). However, we believe our case is unique because our patient had true pump sphincter erosion and was success-fully treated by a conservative surgical procedure based on cleaning and repositioning the pump.

## DISCUSSION AND FUTURE PERSPECTIVES

We report the first case of sphincter pump erosion treated without removing or replacing the sphincter. When we decided to maintain the sphincter, we have considered the local and systemic conditions of the patient. The decision was taken together with the patient who was aware of the risks associated with the presence of a potentially infected sphincter. As we have learned from infected penile prosthesis (6), the conservation of the implant after rinsing it with antibiotics may be a good treatment alternative with more than 80% good results (7). Salvage surgery can also be considered for infected sphincter without erosion (10).

### Take home message

Surgical treatment without sphincter removal can be an option for patients with eroded sphincters. The patient must not present signs of systemic infection and should have good local conditions. Larger series adopting this approach in select cases are needed to validate this alternative.
